# Learning curves and teaching when acquiring nut-cracking in humans and chimpanzees

**DOI:** 10.1038/s41598-018-38392-8

**Published:** 2019-02-06

**Authors:** Christophe Boesch, Daša Bombjaková, Amelia Meier, Roger Mundry

**Affiliations:** 1 Department of Primatology, Max Planck Institute for Evolutionary Anthropology, Deutscher Platz 6, 04103 Leipzig, Germany; 2 Department of Anthropology, University College London, 14 Taviton Street, WC1H 0BW London, UK; 3 Nicholas School of the Environment, Duke University, Durham, North Carolina USA; 4 Max Planck Institute for Evolutionary Anthropology, Deutscher Platz 6, 04103 Leipzig, Germany

## Abstract

Humans are considered superior to other species in their tool using skills. However, most of our knowledge about animals comes from observations in artificial conditions with individuals removed from their natural environment. We present a first comparison of humans and chimpanzees spontaneously acquiring the same technique as they forage in their natural environment. We compared the acquisition of the *Panda* nut-cracking technique between Mbendjele foragers from the Republic of Congo and the Taï chimpanzees from Côte d’Ivoire. Both species initially acquire the technique slowly with similar kinds of mistakes, with years of practice required for the apprentice to become expert. Chimpanzees more rapidly acquired the technique when an apprentice, and reached adult efficiency earlier than humans. Adult efficiencies in both species did not differ significantly. Expert-apprentice interactions showed many similar instances of teaching in both species, with more variability in humans due, in part to their more complex technique. While in humans, teaching occurred both vertically and obliquely, only the former existed in chimpanzees. This comparison of the acquisition of a natural technique clarifies how the two species differed in their technical intelligence. Furthermore, our observations support the idea of teaching in both species being more frequent for difficult skills.

## Introduction

Humans are proposed to possess a unique form of intelligence termed “technical intelligence” which has favored the emergence of specialized skills in thinking, modifying, and combining material objects, as well as using them to modify the outside world to serve their interest^1,2^. The discovery that early in human evolution, people used more complex stone tools than those used by other species provides support for the view that even in early stages of evolution our ancestors possessed elements of technical intelligence that allowed humans to become far more dependent on technology than other species (e.g.^3,4^). Some authors even suggested that certain areas of the brain present morphological specializations that would be supporting this unique technical skill^5,6^. Even if recent observations of wild primate populations document a large diversity of tool use techniques with unexpectedly complex aspects, such as the use of multiple concurrent tools, tool modifications before usage, tool transport, and conditional selection of tools^7–9^, chimpanzees were proposed to use much less complex techniques in the wild than humans^4,10^. A comparison of the chimpanzee and human technical skills could help to specify the differences in the technical intelligence of both species. In a first comparison, we looked at the adult’s performance cracking the hard-shelled *Panda* nuts and showed that even if the two species adopt their own specific solutions to crack the nuts, the adults of both species reach very similar efficiencies at opening the nuts with chimpanzees sometimes being even more efficient^11^. Now in the present paper, we aim to compare the acquisition of this technique in humans and chimpanzees.

Recent studies about the acquisition of technical intelligence skills in humans revealed that apprentices may need many years of practice before reaching adult expertise^12,13^. Despite social exposure to expert tool users’ performances and advice, apprentices only acquire the skills after many years of practice and with slow progress in performance. For example, stone knappers in Langda, New Guinea begin to acquire the technique as adults but appear to encounter difficulties in following the guidance and advice from skilled individuals, as for at least five years, they continue to produce much shorter adze heads employing different strategies than the ones demonstrated to them^14^. A similar pattern has been observed in Khambhat, India, with the acquisition of another type of stone knapping technique, where apprentices pay no attention to some aspects of the technique used by experts, such that their final products are quite different from those of the latter. As a result, high quality beads are produced only after seven to ten years of practice^15^. Similarly, long learning processes have also been documented for the hourly return rate in hunting and honey, palm heart, or tuber gathering among the Ache or the Hiwi^10^, for the reported age of acquisition in different tasks ranging from food and craft production to music and story-telling among the Tsimane of South America^16^, and for the production of knapping stones as tools for hideworking in Ethiopia^17^.

According to the technical intelligence hypothesis, humans possess specialized technical cognitive skills that facilitate the learning of complex tasks: The main cognitive skills mentioned are a more refined understanding of the properties and the function of the tools, along with a much better cross-modal coordination between vision, hands, object, and force, permitting a more precise and controlled manipulation of tools (e.g.^1,2,18,19^). Complex technological skills typically require a very precise selection of the tool material, often combined with a modification of material to become an efficient tool and thereafter some precise and coordinated manipulations of the tool(s) to achieve success, as has been observed for extractive foraging skills like tuber gathering, nut-cracking, or flaking stones^14^. Thus, this proposition stresses the advantage an elaborated cognitive understanding of tools and their manipulation has on the rate of technical task acquisition. Therefore, we would predict humans to learn a complex technical skill more rapidly than chimpanzees. Some data seem to support this proposition, as limitations in some of the cognitive skills mentioned above have been proposed among captive chimpanzees in experimental studies^18,20^.

Alternatively, the life-history hypothesis proposes a slow acquisition of complex technical skills but high adult proficiencies for humans, based on the suggestion of an important dietary shift in our early ancestors concentrating on high-quality, difficult-to-acquire food resources (e.g.^10,21^). This alternative puts a stronger emphasis on the number of different complex techniques to acquire, rather than on the benefit of more elaborate cognition for learning complex techniques^10^. Under this alternative, chimpanzees might acquire one complex skill more rapidly than humans, but, once adult, would be less efficient than humans. This hypothesis however does not specify the number of different complex techniques an individual needs to acquire before we would observe a slowing down in the learning process linked to higher adult performance^10,21^. First, some aspect of this hypothesis sounds contradictory when predicting higher cognitive performance to be associated with elongated learning period for these techniques^10,21^. Second, the test proposed by the authors was based on the assumption that “Daily food acquisition and consumption are virtually the same for chimpanzees from the juvenile period onward.” (10, p.161). However, Taï chimpanzees have been shown to share large amounts of meat after a successful hunt among group members^22,23^. In addition, Taï chimpanzees learn the complex nut-cracking techniques for years, and mothers share large amount of nuts with them^7^. So despite some imprecision, this alternative hypothesis predicts Mbendjele people to learn the nut-cracking technique more slowly than Taï chimpanzees while becoming more efficient once adult.

However, both hypotheses considers mainly complex techniques, and we need to clarify whether the nut-cracking technique is one of them. If, in general, tool use is considered a more complex technique than foraging with bare hands^3,11^, nut-cracking with hammers is considered a more complex technique than single tool techniques requiring no precise aiming movements^18^. Nut cracking necessitates the combination of two tools, the hammer and the anvil, with a third object, the nut; this implies multiple concurrent decisions to execute for success. First, the optimal hammer will change as a function of the availability of the potential hammers, the hardness of the nut to crack, and the type of the anvil^11,24,25^. Second, the transport of a hammer for hard nuts often takes place from a location out-of-sight from the nut-cracking site, necessitating anticipation and planning^25,26^. Third, the modification of an object to produce a hammer is required when no optimal hammer is available in the vicinity, demanding tool-making skills^27^. Fourth, the placement of the *Panda* nuts on the anvil requires precise positioning and repositioning to access each of the three embedded kernels^7^. Fifth, the handling of the hammer requires precise bimanual coordination to aim the nut from the right angle; and sixth, the force applied to the hammer must be controlled to hit the nut with the appropriate impact and prevent smashing it into pieces^18,24,25^. For all these decisions, different alternatives are possible but only a few make success likely, so that nut cracking has been observed in only a few populations of three primate species, and only chimpanzees and humans were seen to crack different species of nuts with flexible tool selection^11,24,27,28^. Hence, we suggest that the nut cracking technique would allow for a good test of the technical intelligence hypothesis.

Very few studies exist that compare humans and chimpanzees performing the same technique in a similar environment. In fact, most studies compared captive living chimpanzees with free ranging and freely socializing humans (e.g.^19,29^). Boesch *et al*^11^. compared humans and chimpanzees in their forested environment spontaneously cracking the same species of very hard nuts, *Panda oleosa*, with hammers. Although the technical challenge is identical when opening *Panda* nuts during forest forays, humans and chimpanzees adopted their own specific way of solving it, with humans using metal bushknives as anvils while chimpanzees use naturally occurring roots or stones as anvils, so that the two technical solutions are not exactly comparable. This reflects the way that both species solve the same technical challenge. To compare the learning process of humans and chimpanzees, we here present a study comparing how Mbendjele children and Taï chimpanzee youngsters acquired the *Panda* nut-cracking skills in their natural environment and within their natural social groups.

## Social dimension of technical skill acquisition

One important aspect of human learning, which is seen in many different societies, is that skill acquisition often occurs in social settings which include group members possessing different levels of expertise^30,31^. Stout (14, p. 702) emphasized this by stating that “… Social scaffolding for the learning process is also provided by the dynamics of the adze-making community itself. Virtually all technical operations in the production of stone adzes are conducted as group activities with a great deal of interactions among individuals”. Hence, naïve individuals are practicing in an environment where most aspects of the technique can be observed whilst performed, and the necessary tools are accessible to them. This dimension is considered to be essential in human skill acquisition, whereby learners induce teaching and teachers instruct learners^30,31^.

Unfortunately, this social dimension of the learning process is often excluded in animal studies in captive settings, thereby preventing subjects from acquiring precious information needed to learn the physical properties of objects and their potential functions (e.g.^20,32^). By depriving young individuals of social stimuli when growing up in a technically active social environment, the naïve individuals might not have the opportunity to develop their prior knowledge about the objects in the environment, nor to see what older group members do with them.

Teaching, that can be defined as “an activity that is pursued in order to increase the knowledge (or understanding) of another who lacks knowledge, has partial knowledge or possesses a false belief”^33^, has been documented extensively in western human societies. With the prominence of school systems, teaching was proposed to be a uniquely human ability in line with the technical intelligence hypothesis^32,34^. However, the limited evidence or even absence of teaching reported for many traditional human societies has raised the question of how typical teaching is for humans^30,31^. From a more evolutionary perspective, some authors have proposed that teaching should be studied when it improves learning of a task that would otherwise be impedingly difficult for learners to achieve^35–37^. In other words, easy skills may be learned without any teaching, whereas hard to acquire skills might induce teaching. Hence, to study teaching we need to consider “what” is transmitted and not only “whether” something is transmitted^38^.

One challenge when comparing pedagogical strategies within and between species is that they will be very skill-specific, and most technical challenges require unique solutions with some strategies benefiting from instruction and others not. Therefore, it could be very misleading and uninformative to compare pedagogical strategies across different skills. For example, fishing for termites requires inserting a stick into the opening of a mound and removing the stick with the termites biting on it - a straightforward technique with no pedagogical interactions observed between mother and infant in Gombe chimpanzees^39^. In contrast, nut cracking requires the subject to bring three different objects together, the nut, the anvil, and the hammer. Each item must possess specific visible and invisible physical properties so that once the correct physical strength is applied, the user can crack open the nuts. This technique requires both physical practice and a good sense for what makes a good tool, and many teaching interactions between mothers and infants have been documented in Taï chimpanzees^7^. Comparing such techniques suggests that Gombe chimpanzees cannot teach whereas Taï chimpanzees can, which would simply be wrong.

In the present analysis, we compared the pedagogical interactions between experts and apprentices during the nut cracking between the Mbendjele children and the Taï chimpanzees. Because of the difficulties researchers have in agreeing on a simple and clear definition of teaching for humans, let alone one that could apply across species^35,40,41^, we recorded all interactions coming from the expert or requested by the apprentice to the experts that include any of the nut-cracking behavior elements^42^. These interactions include facilitating access to tools or nuts, and providing information or correcting errors in the apprentice with or without demonstrations. We based our study on direct observations to prevent ourselves from imposing western pre-conceptions regarding what teaching should look like when using self-reporting interviews^31^.

## Cross-species comparison in skill acquisition

Cross-species comparisons are quite challenging because the species often live in quite different environments where important differences in living conditions, prior knowledge, and ecological conditions prevail^36^. We also must consider that life-history traits differ between species, among which maturation rates, weaning age, age of parturition, and life span can affect the acquisition of skills. Therefore, a direct comparison between species without accounting for life-history traits might be completely misleading^36,43^. Chimpanzee life-history is shorter than that of humans, whereby the former possesses a shorter life span, and matures and reproduces earlier^44,45^.

This point is important as the subadult phase is generally considered as the prime period for learning during their lifespan. As maturation may not follow a linear development over time, there is no single easy way to control for maturation differences between species. Some have suggested correcting for the different life spans observed in the species compared. This way the longer-lived species would be assigned comparatively similar values to those shorter-lived ones^46^. Others have suggested correcting for the different maturation rates of the compared species based on different physiological or morphological markers, such as gestation time or brain development^44^. On the other side, most comparative experimental psychological studies tend to simply ignore this and do not attempt to correct for age of maturation differences among the studied species^44^. By doing so, sequences of developmental stages of different cognitive traits could be compared, while comparison of the rates of appearance of different traits would be misleading.

In our study, we also wanted to account for important population differences regarding maturation within the two species. For example, age of first reproduction varies in traditional human societies from 17.7 years in the Ache to 25.7 years among the Gainj and Asai^47^. Since age of first reproduction is often considered to be one of the main life-history markers^48^, we will present our comparisons between the two species both with absolute age estimates and by correcting age with population-specific age of first reproduction in the Mbendjele (18.5 years for the Aka^47^) and Taï chimpanzees (13.75 years^7^).

## Results

### Number of nuts cracked per minute

**Figure 1:**
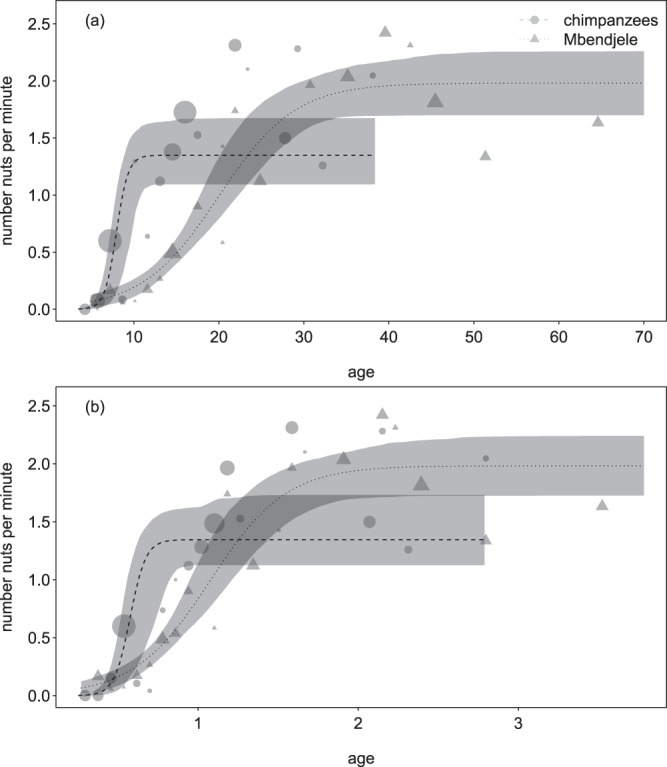
Learning curves for the ‘number of nuts cracked per minute’ in Taï chimpanzees and Mbendjele foragers; (**a**) when considering absolute age (above) and (**b**) when considering relative age whereby 1.0 corresponds to the population-specific age of first reproduction. Indicated are the fitted model and its confidence intervals. For the plot age was binned (bin width: 0.1 year), and the number nuts cracked per minute was averaged per age bin. Symbol area represents the total observation time per age bin (0.1 to 15.8 hours).

For the number of nuts cracked per minute, both models (with absolute and relative age, respectively) revealed the development to reach adult performance earlier in chimpanzees (at an age of about 10 years) as compared to humans (ca. 40 years; Tables 1 and 2; confidence interval, CI, of the interaction between group and age not comprising zero; Fig. 1). Adult performance seemed higher in humans, though the difference was not statistically significant.

**Table 1:** Panda nut-cracking learning curves for the ‘number of nuts cracked per minute’ for Mbendjele foragers and Taï chimpanzees.

Term*	Estimate	Lower CI	Upper CI	Min	Max
Intercept, c_0_	15.576	4.439	36.639	15.472	18.543
Age^a^, c_1_	21.454	9.092	48.030	21.375	24.782
Group^b^, c_2_	−15.879	−37.048	−4.343	−18.786	−15.760
Group:age^c^, c_3_	−18.172	−44.756	−4.505	−21.463	−17.629
Sex^d^, c_4_	0.451	−0.636	4.793	0.283	0.706
Help^e^, c_5_	0.006	−0.496	0.699	−0.053	0.087
Asym^f^, c_6_	1.348	0.873	1.886	1.289	1.413
Group:asym^g^, c_7_	0.632	−0.271	1.307	0.561	0.677

Model results for absolute age and group (human or chimpanzee; indicated are the estimated coefficients, together with confidence limits and the minimum and maximum estimates derived from data dropping individuals one at a time).

*The indexed coefficients (*c*_0_ to *c*_7_) refer to the coefficients as indicated in equation(1) and (2).

^a^z-transformed to a mean of zero and a standard deviation of one; mean (standard deviation) of the original predictor was 18.634 (14.722; years).

^b^humans = 0, chimpanzees = 1.

^c^interaction term informing about how much the effect of age in humans differed from that in chimpanzees.

^d^dummy coded (females = 0, males = 1) and then centered to a mean of zero.

^e^z-transformed to a mean of zero and a standard deviation of one; mean (standard deviation) of the original predictor was 0.061 (0.182; proportion sessions with help present).

^f^fitted asymptotic performance of adult chimpanzees.

^g^interaction term informing about how much the asymptotic performance of humans differed from that of chimpanzees.

**Table 2:** Panda nut-cracking learning curves for the ‘number of nuts cracked per minute’ for Mbendjele foragers and Taï chimpanzees.

Term*	Estimate	Lower CI	Upper CI	Min	Max
Intercept, c_0_	11.371	1.826	25.008	11.026	14.669
Age^a^, c_1_	16.524	5.982	32.836	16.141	20.510
Group^b^, c_2_	−11.136	−24.774	−1.089	−14.283	−10.779
Group:age^c^, c_3_	−13.136	−29.332	−1.625	−16.921	−12.801
Sex^d^, c_4_	0.476	−2.219	4.199	0.325	0.747
Help^e^, c_5_	0.009	−0.784	1.018	−0.049	0.072
Asym^f^, c_6_	1.345	0.957	2.146	1.313	1.422
Group:asym^g^, c_7_	0.637	−0.373	1.190	0.549	0.667

Model results for relative age and group (human or chimpanzee).

*The indexed coefficients (*c*_0_ to *c*_7_) refer to the coefficients as indicated in equation(1) and (2).

^a^z-transformed to a mean of zero and a standard deviation of one; mean (standard deviation) of the original predictor was 1.131 (0.807; fractions of maturation age).

^b^humans = 0, chimpanzees = 1.

^c^interaction term informing about how much the effect of age in humans differed from that in chimpanzees.

^d^dummy coded (females = 0, males = 1) and then centered to a mean of zero.

^e^z-transformed to a mean of zero and a standard deviation of one; mean (standard deviation) of the original predictor was 0.061 (0.182; proportion sessions with help present).

^f^fitted asymptotic performance of adult chimpanzees.

^g^interaction term informing about how much the asymptotic performance of humans differed from that of chimpanzees.

### Probability for a nut to crack per hit

**Figure 2:**
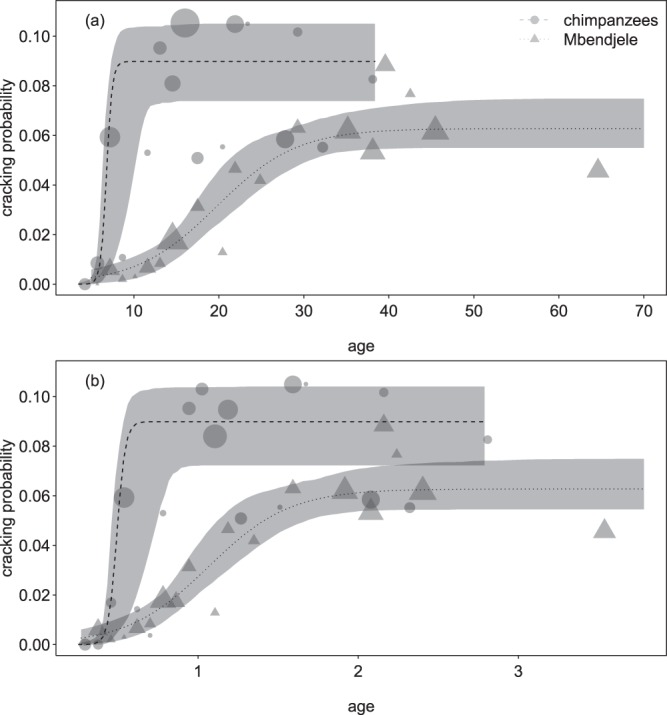
Learning curves for the ‘probability for a nut to crack per hit’ in Taï chimpanzees and Mbendjele foragers; (**a**) when considering absolute age (above) and (**b**) when considering relative age whereby 1.0 corresponds to the population-specific age of first reproduction. Indicated are the fitted model and its confidence intervals. For the plot age was binned (bin width: 0.1 times maturation age) and the probabilities of nuts to be cracked were averaged per age bin. Symbol area represents the total number hits per age bin (2.9 to 180.1 and 2.9 to 272.3 in (**a**,**b**), respectively).

The results for the probability of a strike to successfully crack the nut largely matched the results of the number of nuts cracked per minute: chimpanzees reached adult performance earlier than humans (Tables 3 and 4; Fig. 2). For the cracking probability chimpanzees seemed to have higher adult performance levels, but again the difference was not statistically significant.

**Table 3:** Panda nut-cracking learning curves for the ‘probability for a nut to crack per hit’ for Mbendjele foragers and Taï chimpanzees.

Term*	Estimate	Lower CI	Upper CI	Min	Max
Intercept, c_0_	34.701	4.489	80.663	18.375	47.712
Age^a^, c_1_	43.505	8.247	101.019	24.814	58.153
Group^b^, c_2_	−34.959	−81.025	−3.960	−47.986	−18.686
Group:age^c^, c_3_	−40.394	−98.099	−3.847	−55.357	−21.557
Sex^d^, c_4_	0.377	−1.134	4.725	−0.059	0.910
Help^e^, c_5_	−0.119	−1.136	1.185	−0.170	0.054
Asym^f^, c_6_	0.090	0.066	0.113	0.083	0.094
Group:asym^g^, c_7_	−0.027	−0.053	0.199	−0.031	−0.020

Model results for absolute age and group (human or chimpanzee).

*The indexed coefficients (*c*_0_ to *c*_7_) refer to the coefficients as indicated in equation(1) and (2).

^a^z-transformed to a mean of zero and a standard deviation of one; mean (standard deviation) of the original predictor was 18.523 (14.723; years).

^b^humans = 0, chimpanzees = 1.

^c^interaction term informing about how much the effect of age in humans differed from that in chimpanzees

^d^dummy coded (females = 0, males = 1) and then centered to a mean of zero.

^e^z-transformed to a mean of zero and a standard deviation of one; mean (standard deviation) of the original predictor was 0.064 (0.186; proportion sessions with help present).

^f^fitted asymptotic performance of adult chimpanzees.

^g^interaction term informing about how much the asymptotic performance of humans differed from that of chimpanzees.

**Table 4:** Panda nut-cracking learning curves for the ‘probability for a nut to crack per hit’ for Mbendjele foragers and Taï chimpanzees.

Term*	Estimate	Lower CI	Upper CI	Min	Max
Intercept, c_0_	24.172	2.325	65.901	13.164	37.444
Age^a^, c_1_	31.001	6.741	85.989	18.677	46.071
Group^b^, c_2_	−23.933	−65.497	−1.993	−37.278	−12.962
Group:age^c^, c_3_	−27.810	−82.607	−1.343	−43.194	−15.354
Sex^d^, c_4_	0.401	−1.201	4.405	−0.039	0.946
Help^e^, c_5_	−0.127	−0.980	1.187	−0.173	0.056
Asym^f^, c_6_	0.090	0.060	0.111	0.083	0.095
Group:asym^g^, c_7_	−0.027	−0.050	0.017	−0.032	−0.020

Model results for relative age and group (human or chimpanzee).

*The indexed coefficients (*c*_0_ to *c*_7_) refer to the coefficients as indicated in equation(1) and.(2)

^a^z-transformed to a mean of zero and a standard deviation of one; mean (standard deviation) of the original predictor was 1.126 (0.811; fractions of maturation age).

^b^humans = 0, chimpanzees = 1.

^c^interaction term informing about how much the effect of age in humans differed from that in chimpanzees.

^d^dummy coded (females = 0, males = 1) and then centered to a mean of zero.

^e^z-transformed to a mean of zero and a standard deviation of one; mean (standard deviation) of the original predictor was 0.064 (0.186; proportion sessions with help present).

^f^fitted asymptotic performance of adult chimpanzees.

^g^interaction term informing about how much the asymptotic performance of humans differed from that of chimpanzees.

### Expert-apprentice interactions in the nut-cracking context

Table 5 presents the ethogram of the different types of teaching interventions observed in the Mbendjele and chimpanzees in the context of cracking the hard *Panda* nuts. In both species, expert interventions were observed regularly in the context of nut cracking. Those related to taking tools or nuts from the young were excluded by us from the teaching interactions (Table 5; “take tool”). For the Mbendjele we recorded 83 teaching events of 34 different types observed during 1,617 minutes of nut-cracking involving an apprentice (see Table 5). For Taï chimpanzees, our observation duration was 4,137 minutes and we recorded mothers to successfully stimulate the use of the hammer in 157 instances, facilitate access to their hammers in 272 occasions, and provide intact nuts for the young to crack on 18 occasions (42, p. 531). In general, among the Mbendjele, expert help was observed every 20 minutes of nut cracking by the apprentice, whereas in the Taï chimpanzees it was observed every 10 minutes.

**Table 5:** Ethogram of the different teaching interaction types observed between expert and apprentice when nut cracking among the Mbendjele forest foragers and the Taï chimpanzees.

Technical aspect	Definition	Pedagogic intervention types	Variations	Examples	Pedagogic intervention types seen in chimpanzee
Choose a nut	Expert selects an intact nut for the youngster.	1- Expert tells the youngster to change nut before continuing to try to open one, 2- Expert selects one good nut from the pile in front of them and hold it for the youngster to take and place, 3- Expert selects a good nut, either “shows nut placement” to the youngster, or “places the nut” on the blade for the youngster, 4- For a very young cracker, expert selects a partially opened nut for the young to crack.		4- Young girl (12 y) did so with boy (5 y).	3- Mothers leaves some of her collected nuts at the anvil for the youngster to crack
Show nut placement	Expert shows with its hand the spot on the nut that needs to be placed against the blade of the anvil.	1- Expert points directly at the correct spot on the nut that is placed on the blade of bushknife, 2- Expert follows with a finger the dehiscent lines on the nut to highlight the positioning of the nut, 3- Previous is accompanied with the verbal expression “go from the head”, 4- Expert points to the correct spot on an intact nut held in its hand while the youngster tries to place its own nut, 5- Young expert can ask an adult expert for the position on the nut and then show it to the naive that is cracking.	* Adult points by touching the spot on the nut or from farther away, * Adult points either with the forefinger, the hammer or the knife.	3- Mother does that nicely with her daughter (11 y). 5- Daughter (10 y) did ask her mother the positioning of the nut before showing it to boy (8 y).	**Not seen**
Place the nut	Expert takes the nut hit by the youngster and replaces it correctly on the bushknife blade.	1- Expert places the nut on the blade for the youngster to hold it in the same position, 2- Expert holds the nut in place on the blade during the first 3 to 4 hits of the youngster, the later often places his finger above the ones of adult holding the nut, 3- Expert shows the placement of the nut on the youngster’s bushknife, and then places it on their bushknife to open the nut.	* Youngster often places its hand above the expert hand as it holds the nut. * Some verbal explanation can accompany this action.	4- Young woman (18 y) does it with younger girl (11 y).	1- Salomé, and Héra replaced correctly a nut already placed by their sons
Show the anvil placement	Expert points with the finger to the correct place on the blade of the anvil	1- Expert points at the absence of a support that should be corrected 2- Expert provides a piece of wood to support the bushknife on ground, and sometimes places the piece under the bushknife		1- Mother says to daughter “get a support wood” 2- Mother throws a small hammer as support at daughter (8 y).	**NA**
Helps with the anvil	Expert intervenes to modify the use or selection of the anvil by the youngster.	1- Expert helps with the hand in supporting the anvil in a good position, 2- Expert corrects the placement of the anvil on the support, 3- Expert suggest to change the anvil, especially for badly worned bushknife, 4- Expert gives its own axe for the youngster to use.		4- Seen only once with mother giving axe to daughter (12 y).	**NA**
Helps with the hammer	Expert intervenes to modify the use or selection of the hammer used by the youngster.	1-Expert points at what length the youngster may cut the branch to make a useable hammer, 2- Expert may suggest verbally to change hammer either too long or too thick, 3- Expert correct the length of youngster’s hammer itself by cutting it with a bushknife.		1: Young woman (16 y) did so with another woman equally skilled as her. 3: Mother did this with her son (15 y).	a- Mothers provide their hammer only or with some intact nuts to the youngster b- Mothers may place the hammer and nuts correctly.
Helps with the extraction of the kernel	Expert shows the base of the exposed almond that should be cut to free it from the opened shell of the nut.	1- Expert points with its finger to the base of the kernel where it is fixed to the shell held in the hand by the youngster, 2- Expert takes the nut in its hand and indicates with its fingers the way to extract the kernel, 3- Expert extracts with a knife the kernel from an open shell to show how to detach the kernel to the youngster.		Adults were seen to do this with seven young individuals.	**NA**
Demonstration	Expert performs whole or parts of the actions of nut cracking with the tools used by the youngster.	1- Expert places itself in the back of the youngster, and without the later moving takes his nut and his hammer and cracks the nuts in front of the youngster, while the later keeps the bushknife (or axe) stable, 2- Expert places itself on the side of the youngster and does the same without the youngster moving from its position, 3- Expert can just give some hits on the nut placed on the youngster’s blade and let him continue or crack so one to a few nuts, 4- Expert uses it own hammer to crack the youngster nut on the youngster anvil.	In all cases, the youngster does not move from its position, keeps the anvil stable and continues to crack with its tool once the expert is finished.	1: Mother did so 3 times with her son (15 y). 2: Mother cracked 4 nuts with son (15 y). 3: Father did so with his son, 4: Young woman (18 y) did so with girl (11 y) as she was sitting in front of her.	2- Ricci demonstrates nut cracking and opens 3 nuts with her daughter.
Correct body position or movement	Expert manually or verbally explains a mistake in the movements of the youngster.	1- Expert physically takes part of the body of the youngster and replace it so as to adopt the correct position (e.g.: sitting, stabilizing bushknife,…), 2- Expert explains verbally the correct position of movement to perform (often accompanied with a movement of the adult).	The provided verbal comments may be followed or ignored by the youngster	1: Mother changes legs position of girl (12 y), but she resumes previous position to crack.	**Not seen**
Ask for help	Youngster asks for advise toward an expert.	1- Youngster hold the whole or part of the nut towards the expert and ask a question, 2- Youngster in addition to the above indicate to expert with its forefinger a spot or a line on the nut, 3- Youngster show how he places the nut on the blade to an expert looking for its response, 4- Youngster ask for a tool, bushknife, support or hammer.	* Some youngster’s demands are simply ignored. * Negative answers are given (e.g. “Open your eyes”, “you are lazy” or gestures like hand-waves).		1- Youngsters facing difficulties would whimper looking at their mother 4- Youngsters ask for the mother’s hammer with the extended hand
Take tool	Adult takes away a tool used by the youngster	1- Adult takes one of the tool used by the youngster away, either the hammer, the bushknife, axe or the support, 2- Adult asks for the tool used by the youngster and the later obey, 3- Adult asks for a tool to the youngster that interrupts itself to provide the expert with it.	In all case, the adult were successful, and the youngster accepted without visible negative response.		1- Adults take hammer and anvil from youngsters regularly

For each technical aspect of the nut-cracking technique, we describe the different interaction variations observed in the Mbendjele, with comments about possible modifications of these variations and a few examples numbered according to variation (with indications about the age of the individuals). The final column highlights whether or not the teaching interaction was observed in Taï chimpanzees. If the interaction was equivalent to that seen with the Mbendjele, we maintained the numbering for each technical variation, if similar but somehow divergent, the variation was assigned a letter.

Of the total of 34 different types of teaching interactions, 9 occurred in relation to aspects not present in the chimpanzee nut-cracking technique, namely related to the placement of a mobile anvil, and the kernel extraction by a third party (“NA” in Table 5). Additionally, 5 of the types were verbal interactions from the expert that are not available to chimpanzees. Of the 20 teaching interactions observed in the Mbendjele that chimpanzees could have accomplished, only 7 types were seen in chimpanzees. Two technical aspects not subject to teaching in chimpanzees were directions regarding the body position of the apprentice and how to place the nut correctly on the cutting blade. The eleven remaining uniquely human teaching interactions pertained to experts molding the apprentice’s body or movement, pointing to or demonstrating the correct way to perform the activity.

Teaching interactions in the Mbendjele were directed vertically to learners by mothers (N = 17), fathers (N = 3), and an aunt (N = 1), or horizontally from older to younger siblings (N = 1) or from older to younger cousins (N = 3) and regularly from more distantly related individuals (N = 14). Whereas in Taï chimpanzees, all teaching interactions were directly from the mothers to the offspring (N = 447). Despite the diversity of teaching interactions in either species, there was no immediate effect of expert interventions on the progress of the nut-cracking learning curves in either species (see Tables 1–4).

## Discussion

The present study of two species, humans and chimpanzees, is the first to compare the same technical task naturally performed during daily forays in the forest, and with youngsters regularly exposed to the technique performed by family and group members. Because of this, both groups were able to build their prior knowledge and experience of the technique from their natural group-specific social experience. Our results illustrate how the two species solve the same technically complex challenge in their own species-specific way and how they acquire the technique. The technical solution of humans is more complex than the solution adopted by the chimpanzees^11^, and we see that children acquired the technique more slowly than chimpanzees under the two efficiency measures that we used. Thus, this comparison does not support the predictions arising from the technical intelligence hypothesis which, based on some unique human cognitive skills and experimental studies using captive chimpanzees^2,20,32^, proposed that humans would be able to solve the technical challenges of nut-cracking more rapidly than chimpanzees. The life-history hypothesis that predicted slower learning during the juvenile period for extended periods, combined with higher adult efficiencies in humans compared to chimpanzees^10^ was not supported either, as adult’s efficiency tended to be lower in humans than in chimpanzees for the probability for a nut to crack per hit. This unexpected result requires a more careful discussion of the evolutionary process leading to the tested predictions.

From an evolutionary point of view, it should not come as a surprise that populations facing similar ecological and technical challenges over time develop similarly efficient solutions. Such convergent evolutionary processes have been demonstrated in many different taxa and with species quite distant on the evolutionary scale^49,50^. Chimpanzees and humans are closely related species and therefore such similarity could have been expected. Many aspects of tool use described in both species are similar, such as flexible selection of tools, the presence of tool kits, tool manufacturing, and tool transport. This all points to the fact that the ability to use tools is shared by descent between the two species and therefore similarities in the acquisition of a similar technique are expected.

### Learning curve of nut cracking

In general, the maturation of humans is presented as extending over a longer time period than in any other non-human primate, allowing for longer learning periods, which in turn could explain why humans are able to learn many more and much more complex techniques^51,52^. In this sense, the fact that we observed quite similar sigmoidal learning curves in the two species reveals flexibility when learning a complex technical skill. Unexpectedly, when a difference did appear, it was that the young chimpanzees progressed more rapidly and that the difference in learning curves persisted even after we corrected for the differences in rate of physical maturation between the two species.

One prediction of the life-history hypothesis is that children would acquire the technique slowly but continue to improve as adults to higher efficiencies than chimpanzees. Even though the first part of the prediction is supported by our study, the second part is contradicted by our results for the adult performance in both species (see also^11^). This could suggest that the cognitive improvements predicted by the technical intelligence hypothesis are not enough to compensate for the additional number of different techniques children need to learn, especially during the period between 2 to 7 years of age. Kaplan and Robens^21^ suggested three phases by which foraging challenges could lead to higher intelligence and longevity, the last one concerning humans. In this framework, our result would suggest that the second phase, proposed to be present in the common ancestor of chimpanzees and humans, when a specialization in extracting high quality foods emerges, would produce flexibilities sufficient to acquire the cognitive skills for nut cracking with tools. Thus, when comparing chimpanzees and humans on only one technique, chimpanzees could outcompete humans, whereas when incorporating all the skills learned by the young Mbendjele, chimpanzees would fare poorly.

The selective advantage of learning a complex technique, would however, not be compensated by higher cognitive understanding of the tools for one specific skill, but rather at a general level so as to allow more flexible solutions to an array of tasks. The adult comparisons showed that Taï females were especially good with heavy stone hammers compared to Mbendjele women using a bushknife, suggesting a specialization in the manipulation of heavy tools in chimpanzees that make them outperform humans. However, the bushknife in the Mbendjele is a multifunctional tool *par excellence* used in dozens of different contexts and purposes. The relative paucity of multifunctional tools in chimpanzees^36^ could be an indication of how more precisely the technical intelligence hypothesis would work: by selecting for flexible uses of a few generalized tools, humans were able to enlarge and improve their foraging success with different hard-to-access food resources.

For complex technical skills, such as nut cracking, both species follow years-long apprenticeships with a very similar learning curve. They initially progress slowly as the young apprentices first learn how to combine the required objects - nut, anvil and hammer - and then they gradually learn to master the mechanical challenges of adequately using these objects. A minimum of over 10 years of practice is necessary for both the Mbendjele and the Taï chimpanzees before an apprentice reaches the same level of efficiency as adults.

Furthermore, with the Mbendjele, we observed that depending on the opportunities for practice available to them, some women learned the task very late in life, namely when they already had children. For example, one 25-year-old woman with a 2-year-old baby did not have much practice with the tools, and cracking nuts still represented a challenge for her, as she encountered problems with the stability of the nuts. She dropped the nut 107 times for the 88 first nuts of the season due to fear of hitting her fingers, and she still did so eleven times - twice cutting her fingers and bleeding to the point where she interrupted her nut cracking. In the Mbendjele, such insecurities about holding the nuts were typical of 12 to 15 years old apprentices. This illustrates the larger impact practice can have on an individual’s learning curve independent of age. As the tools are often limited to one per family, the practice of apprentices can conflict directly with the foraging efficiency of their mothers. Alternatively, cultural norms could influence individuals to choose to specialize in different skills. Mbendjele refer to people’s specializations in terms of guardianship, as “domains of interest to be looked after” (èkóndʒà). This means that not all individuals choose to specialize in nut-cracking skills. Finally, nuts could be a more important food source to the chimpanzees than it is for the Mbendjele, resulting in less practice among the latter. However, our observations of the Taï chimpanzees indicate that they eat the *Panda* nuts quite rarely.

The slow learning of the Mbendjele relating to nut cracking is very reminiscent of the long learning process described for stone knapping in other societies^10,14,15^, where approximately 10 years of practice are needed to reach adult expertise. This suggests that the technical challenges faced by apprentices to master nut cracking are similar to the ones faced by apprentices when stone knapping. Indeed, in both cases, the technique requires an integration of motor control during a forceful physical movement with relatively large objects (the mastering of adequate force applications) and the notion of the correct angle of impact. In both cases, humans require an extended period of practice to master the skill. Intriguingly, chimpanzees with a different morphology and a different technique are mastering the challenges in a similar way to humans. Both species start to show an interest in the tools and the nut-cracking behavior before 5 years of age, and they make similar mistakes such as hitting the anvil without a nut, switching nuts when not successful, or hitting the ground with the hammer. Thus, at this age, cognitive differences affecting the learning of nut cracking in the two species are not evident from our observations.

The more rapid progress seen in chimpanzees compared to humans might reveal a quicker adaptation of chimpanzees to the technical challenges of the task. This may in part be due to the greater selection pressures on chimpanzees to use their hands and arms in their daily life. From a very early age, they walk, climb trees, and pull or push branches to negotiate their way through the forest despite their relatively large body size, as well as prepare food from hard fruits. Mistakes 25 meters high in a tree can be deadly, and therefore young chimpanzees are under strong selective pressure to develop rapid control of motor performance with their hands and arms. In comparison, for humans, hands and arms are less important early in life and we might expect that motor control with large objects is less advanced than in chimpanzees.

The more rapid progress of chimpanzees might also reflect the more complex bimanual coordination that the specific Mbendjele nut-cracking technique is imposing on the nutcracker^11^. Where chimpanzees precisely balance the *Panda* nuts on the fixed anvil, and thereafter can use one or both hands to strike the nut with the hammer, the Mbendjele have to constantly hold the nuts in a precise position on the blade of the bushknife while the other hand strikes the nut with the hammer. The latter technique requires more precision as the stability of the bushknife is dependent on the skills of the nutcracker, so the potential for the nut to slip off the blade is high when hit. If this happens, the fingers holding the nut are at risk of being cut by the sharp blade of the bushknife (we saw this happening about 21 times). To limit this risk, the children tend to hold the nut more loosely, increasing the likelihood that the nut slips or jumps away when hit (N = 176 times). Alternatively, they may hit the nut with less force, increasing the number of hits required to open the nut. Therefore, the huge advantage of using a bushknife to exploit a greater range of nut species^11^ comes with the cost that it is more difficult to learn the technique.

### Teaching in humans and chimpanzees

Our observations highlight the fact that the complexity of the technical skills required for nut-cracking are great enough to favor the emergence of teaching as a means of helping the apprentice to acquire the technique. In both species learning the technique required 10 or more years, so it makes sense for the mothers to speed up the learning process of her young and ensure they reach a certain level of efficiency before the next offspring arrives^36,37^. In addition to our observations of the Mbendjele, we also saw regular pedagogic interactions, for *Panda* and *Irvingia* nut cracking in the Aka of Ndele in the Central African Republic (Boesch pers. obs.). Our observations are in agreement with the suggestion that teaching skills are present in hunter-gatherer societies^37,53^. To teach their young the nut-cracking technique, the Mbendjele and Taï chimpanzees employed seven identical or highly similar teaching interventions (Table 5). However, they differed in expert-apprentice relationships: in chimpanzees teaching interactions were only observed from mothers to offspring; whereas mother-offspring interactions only represented 21 out of 39 teaching interactions among the Mbendjele.

Boyette^54,55^ compared broad patterns of teaching in the neighboring Aka forest foraging people, which he found to occur much less frequently than other types of social learning. In the Aka, teaching in all contexts occurred about 0.017 times per minute, whereas in the Mbendjele, teaching in the nut-cracking context was three times more frequent, occurring about 0.05 times per minute. The rate of teaching for the Mbendjele was quite similar to that observed within a Maya community weaving carpet, in which teaching instances over all contexts, involving many technical ones, occurred about 0.07 times per minute^56^. Among the Mbendjele almost all teaching interactions took the form of “instruction” (in the sense defined in^54^), while in the Aka instruction represented only 8% of the teaching events recorded. This suggests a much higher rate of instructions for nut cracking compared to other contexts. We might have missed some of the commands as we intentionally adopted a cross-species comparative approach and therefore concentrated on the behavioral aspects of teaching. At the same time, it seems likely that a command, defined as the use of directive or joint social activities^54,55^, would not be the most efficient way to help an apprentice in solving a solitary technical task. The difference in teaching frequencies provides some support for the idea that teaching becomes more frequent when the technical challenge of the task increases^11^.

From our direct observations of the Mbendjele, it seems that, for nut cracking to be efficient, the technical difficulties involved in learning might select for elaborate forms of teaching. As an example, a mother demonstrated to her 12-year-old son how to place the nut on the anvil by sitting behind him, and, with her arms around him, she took his hammer in one hand and the *Panda* nut in the other. Then, while talking to her son, who was still sitting in the position he used to crack the nuts, she presented the dehiscent line of the nut to him, placed the nut carefully on the bushknife (still held by her son) and cracked 3 nuts as a demonstration. Then, her son took back his hammer and carefully placed a nut on the blade as explained to him but failed to open it. As a consequence, the mother repeated the same process a second time. The son cracked some more nuts with limited success before stopping. Interestingly, his younger sister then took his tools and tried to crack nuts with even less success. Seeing this, he showed her the dehiscent line and how to place the nut correctly. We saw more examples of these elaborate demonstrations instructing about the position of the nut as well as the position of one’s body to solve technical challenges regarding specific hammers. We also saw similar pedagogic interactions when children were cracking the *Irvingia* nuts^11^.

Our observations of teaching interactions in humans and chimpanzees add new aspects to our knowledge about the teaching abilities in both species. In addition, our observations support an evolutionary approach whereby the emergence of teaching is expected only when it can provide a benefit that could not be acquired by individual or social learning alone^36,37^. This approach expects that teaching should be observed in different animal species, and that the degree of sophistication of the teaching interactions increases as the benefits to the learner increase in the context of a given technique. Supporting this expectation, teaching has been reported in animal species known for their cognitive skills, such as dolphins, carnivores, and certain primates^40^. However, the frequency and type of teaching interactions varies extensively across species, while teaching interactions in which the expert accounts for the apprentice’s level of knowledge and reacts to specific mistakes are limited to a few species. Humans have broadened teaching to different types of horizontal teaching interactions such as between peers, whereas this is absent in chimpanzees as far as we know. The acquisition of language allows humans to give much more precise guidance, while at the same time allowing for mocking comments on the apprentice’s skills. Such forms of verbal reinforcement were considerably more successful in the transmission of, e.g., stone knapping skills than observation alone^55,57^.

In agreement with the previous observations on the limited effect of teaching interactions on the learning curve of the apprentice^14,15^, we did not see any statistical effect of the presence of teaching on the immediate nut-cracking performance. Based on a Western conception that teaching must result in an improvement, this has been proposed as a criterion for teaching^35^. Our observations of teaching in the context of nut cracking did not fulfill this requirement in the short-term, although the teachers clearly intended to transfer information to the apprentices. In addition, for stone knapping it has been argued that individual practice is essential to assimilate the social observations and teaching information on the apprentice’s motoric and physical movements^14^. We argue that knowledge of how a movement has to be enacted does not immediately lead to improvement of that movement until all the physical knowledge is fully integrated through extensive practice.

In conclusion, the comparison of the acquisition of nut-cracking showed that both chimpanzees and humans learn the skill in a very similar way, that the learning is a years- long process, and that experts regularly provide information to apprentices during this process. The unexpected observation that chimpanzees learned the nut cracking more rapidly and reached adult performance earlier than humans does not support the predictions of the technical intelligence nor the life history hypothesis. This is the first comparison of the acquisition of a technical skill habitually performed by the two species as they forage in their natural environment. It provides important new insights about potential differences in the two species and highlights the importance of making cross-species comparisons in situations that are directly comparable.

## Methods

### The subjects

The chimpanzees observed for this study live in the Taï National Park in western Côte d’Ivoire. Their nut-cracking behavior was studied in detail between 1987 and 1993^7^. The population cracks 5 different nut species, including the hard nut, *Panda oleosa*, which is the nut species used in the present study. A detailed analysis of young chimpanzees learning the nut-cracking technique was presented in 8 and this data set will be used for the present comparison. All individuals in the community have been identified since 1982 and were fully habituated to human observers within the following three years, allowing us to know the precise age of the younger individuals. For older individuals, age was estimated based on physical and behavioral characteristics as well as by previous experience from aging individuals with the Gombe chimpanzees (see 7 for more details about the aging procedures).

The Mbendjele foragers live in the Northern part of the Republic of Congo. Our research area was situated near the village of Djoubé on the Motaba River (see 11 for more details). They maintained a hunting and gathering way-of-life, spending long periods in temporary camps gathering natural fruits, nuts, honey, and fishes in the forest. *Panda oleosa* trees were very abundant near the temporary camps providing us with the unique opportunity to follow nut cracking skill acquisition in the children, as we did with the Taï chimpanzees. Aging children, as in all non-literate societies, can be challenging and we based our estimates on the years-long work of DB with this group as she was able to fluently communicate with them to improve the age estimates of younger individuals. For adults, the age estimation has to be considered to become less precise the older they have been estimated. In this paper, we use the term “young” and “youngster” to refer to inexperienced individuals, chimpanzee or Mbendjele, who are learning the nut cracking techniques.

### Panda nut cracking

The *Panda* nuts are one of the hardest nut species growing in Africa, requiring about 16 tons of weight to crack open^58^. *Panda oleosa* trees produce a few hundred nuts each year, but are relatively rare and dispersed in the forest. The shell of the nut is very resistant causing the kernels to remain edible months after having fallen to the ground. The nut contains 3 to 4 kernels individually embedded within the hard wood of the shell. Chimpanzees crack the nuts by balancing them on a root used as an anvil, and pounding them with a heavy stone hammer with repeated hits. Stone hammers vary in weight from 0.5 kg to over 18 kg with the majority of them between 3 kg and 9 kg^59^. The Mbendjele people mostly use a bushknife and more rarely use an axe as anvils, held on the ground with their foot so that the cutting blade is pointing upwards^11^. Then, with one hand, they hold the *Panda* nut in place on the blade and, with their other hand hit it with a light wooden hammer made from the wood of an abundant sapling.

Two strategies are used to access the kernels (see 11 for more details). Chimpanzees place the nuts so that the maximum kinetic energy from the hammer is placed between the two dehiscent lines and the opercula opens under the force leaving large parts of the kernel intact. Imprecise hits risk smashing the whole nut, requiring much more time to sort edible bits from the shell. Powerful hits are used first to break the nut open, and subsequently very soft hits are applied by the chimpanzees, after repositioning carefully the nut, in an attempt to access each individual kernels with the least damage. Only skilled nut-crackers are able to extract some of the kernels intact, the majority of them being cut in one or two pieces as the shell was broken apart. Alternatively, the Mbendjele place the base of the opercula of each kernel precisely on the sharp cutting blade of the bushknife and then hit the nut with a wooden hammer until the blade cuts the whole opercula loose, exposing the intact kernel. They repeat this until they have exposed all the kernels and then place the open nuts on a large *Marantaceae sp*. leaf for another group member to cut free each kernel with a knife and place them in a basket for later consumption at camp as part of a meal. The use of the cutting blade and precise positioning of the nut allows for intact extraction of almost all kernels. Apprentices of both species are, however, still far from the ideal and cut often the kernels in smaller pieces than experts.

### Data collection procedures

In the Djoubé forest, the *Panda* nut production seemed especially good in early 2014. Data collection occurred between May and August 2014, when women had access to edible nuts laying on the ground. One month was spent training on data collection methods to ensure good inter-observer reliability. Video recordings were used whenever more than one individual was involved in nut cracking and any time young were attempting the technique. In our two study populations, we recorded nut-cracking instances in the forest with a focal sampling method^59^. Individuals were observed for a minimum of 20 minutes or until they stopped.

As reported in our previous study^11^, the Mbendjele use a metal tool, a bushknife or axe, as an anvil to crack the nuts, and each family normally had one of each. Collecting data on the children with the Mbendjele proved to be more difficult than expected as independent children rarely followed their mothers in the forest. In addition, if children wanted to crack nuts, they needed the mother’s tool that could potentially limit the mother’s foraging return, and consequently mothers were somehow reluctant to share their tool for long. Nevertheless, we succeeded in collecting 43 nut sequences with children of different ages whenever they followed their mothers in the forest.

Realizing that we would not be able to collect enough data on the children in the forest, we collected *Panda* nuts in the forest at the end of the season and brought them back to camp for the children to crack. We did this only after their mothers had returned to camp from foraging in the forest. That way the children not only obtained an opportunity to crack the nuts if they wanted, but also gained access to the family’s metal tools without affecting their mother’s foraging activities (N = 44 sequences). Cracking the nuts in camp could be easier than in the forest. The ground in camp had been cleared from leaves and branches littering the forest floor, making positioning the bushknife potentially easier. However, we did not gain the impression that this affected performance and this would actually favor humans in the present comparison.

### Nut-cracking efficiency measures

To follow the acquisition of nut-cracking, we used two measures previously developed to study Taï chimpanzee nut-cracking behavior: the “number of hits to open a nut” and “the number of nuts eaten per minute”^11^. The first measure reflects not only the efficiency of the tools selected, as we showed that heavier stone hammers allow the user to open nuts with fewer hits, it also accounts for the dexterity of the nut-cracker to manipulate the hammer. The chimpanzees eat each kernel as they expose them, while the Mbendjele carry the nuts back to camp before consumption. To account for this difference, we compared only the time needed to open the nuts in both species (see as well^11^). Furthermore, to allow for an ontogenetic study we transformed the first measure, as young of both species have been seen attempting to open the nuts for many months and sometimes even years without successfully opening any. Therefore, the first measure was transformed into the “probability for a nut to crack per hit”.

### Teaching - Expert interventions in the nut-cracking

Maternal as well as other group members’ interventions in apprentice nut-cracking attempts were recorded in a continuous fashion with information about the context and the actions as precisely as possible. Since the data for the chimpanzees were collected some 25 years earlier, we used the same behavioral elements we observed between mothers and offspring in the Taï chimpanzees^7,42^. Expert-apprentice interactions occurring before June were reported on site and after June were analyzed from the video recordings following the same ethogram. Videos were also used to transcribe the oral instructions and comments exchanged between expert and apprentice. CB recorded the interactions directly in the forest when recording the nut cracking sequences. AM, who stayed longer in the forest, recorded on a video all children nut-cracking sequences. Later, CB did the coding of the videos including all expert-apprentice interactions, while DB did all the translations of the vocal exchanges occurring between expert and apprentice.

We expected to see new elements compared to what we saw in chimpanzees and therefore kept an open mind to detect all possible variations in the interventions coming from experts. As our focus was of a comparative dimension, we concentrated on non-vocal elements and used clear behavioral operational descriptions of the observations. If spoken instructions, comments or remarks were produced by the experts at the same time, they were recorded and considered only if they were directly related to the expert-apprentice nut-cracking interactions.

### Statistical analysis

Since a standard Generalized Linear Model^60^ cannot model the obviously non-linear (i.e., most likely asymptotic or sigmoidal) nature of the ontogenetic trajectory, we fitted a Generalized non-linear Model. This modeled the effect of age, group, and their interaction as key terms but also included sex and the presence of help on the number of nuts cracked per unit time. Furthermore, we included the observation duration as an offset term^60^. The model was fitted using the following equations:

$${\rm{Number}}\,{\rm{nuts}}={\rm{sigmoidal}}\,\times \,{\rm{obs}}.\,{\rm{time}}\,\times \,({{\rm{c}}}_{6}+{{\rm{c}}}_{7}\times \,{\rm{group}})$$

$${\rm{Sigmoidal}}=\frac{{{\rm{e}}}^{{\rm{c}}0+{\rm{c}}1\times {\rm{group}}+{\rm{c}}2\times {\rm{age}}+{\rm{c}}3\times {\rm{groupxage}}+{\rm{c}}4\times {\rm{sex}}+{\rm{c}}5\times {\rm{help}}}}{1+{{\rm{e}}}^{{\rm{c}}0+{\rm{c}}1\times {\rm{group}}+{\rm{c}}2\times {\rm{age}}+{\rm{c}}3\times {\rm{groupxage}}+{\rm{c}}4\times {\rm{sex}}+{\rm{c}}5\times {\rm{help}}}}$$

where group and sex are both dummy variables being 0 for chimpanzees and females and 1 for Mbendjele and males, respectively. The sigmoidal equation accounts for the sigmoidal nature of ontogenetic trajectories, bounds the fitted values to values larger than zero, and allows the shapes of the trajectories to differ between humans and chimpanzees. The last part (*c*_6_ + *c*_7_ × group) allows for the asymptotic performance to vary between humans and chimpanzees. The coefficients *c*_0_, *c*_2_, and *c*_6_ express the steepness of the increase and when the steepest increase takes place (*c*_0_ and *c*_2_) as well as the (asymptotic) adult performance (*c*_6_) for chimpanzees, and the coefficients *c*_1_, *c*_3_, and *c*_7_ express how these features of the ontogenetic trajectory differed between humans and chimpanzees (the coefficients *c*_4_ and *c*_5_ express the effects of sex and presence of help, respectively). We then used maximum the likelihood to determine the estimated coefficients (*c*_0_ to *c*_7_) best explaining the observed number of cracked nuts (assuming a negative binomial error distribution). We assumed two independent values of theta, the dispersion parameter^61^, for Mbendjele and chimpanzees to account for the possibility of extra variation between them. To avoid pseudo-replication^62^ we aggregated the data per individual and fitted the model to the aggregated data. The model was fitted using the R-function ‘optim’^63^. The stability of the models was good as indicated by the range of estimates obtained when excluding individuals one at a time. We determined 95% confidence intervals by means of a non-parametric bootstrap (using the individuals as sampling units). The model was fitted for the absolute and the relative age. We fitted two essentially identical models (with absolute and relative age, respectively, as predictors) for the probability of an individual strike to crack the nut. These models lacked the offset term for the observation time and were implemented with binomial error structure. The sample sizes were 83 and 79 for the negative binomial and binomial model, respectively.

Several of the full or null models (lacking group and its interactions) did not converge, even when we increased the maximum number of iterations or altered the optimizer. However, stability estimates, confidence intervals, and plots of the data and models suggest that the results can be trusted nevertheless. Since non-converging models preclude likelihood ratio tests we based inference on confidence intervals. We considered an estimate to be significant (at an α of 0.05) when its 95% confidence interval did not encompass zero.

### Ethics statement

The data collected for this study were strictly non-invasive and were approved by the Ethical Board of the Max Planck Society. As such, the study was conducted in accordance with Germany’s law and the rules and regulations governing animal and human research in the European Union. The Mbendjele people had been fully informed about the aim and the methods of the project and provided their informed consent for being included in our study and for the observers to follow them during their natural forays in the forest. For all participants under the age of 18 years old, a direct parent has provided an informed consent. In each African country where field data were collected, all relevant permissions were first obtained from the country’s governmental institutions before starting data collection.
